# Prof. Philip H. Austen

**Published:** 1879-04

**Authors:** Elisha G. Tucker, Jacob L. Williams, Edward N. Harris

**Affiliations:** President; Vice President.; Cor. Secretary.


*Prof. Philip H. Austen, D. D. S., M. D.*—At the monthly meeting of the American Academy of Dental Science, held in Boston, February 5th., 1879, the following resolutions were adopted:

*Resolved*, That the Academy have heard with deep sorrow of the death of one of its Honorary Members, Prof. P. H. Austen, of Baltimore, and in recognition of his character and ability, we desire to express our regret for his loss and our appreciation of his worth.

*Resolved*, That our Academy, and the profession of dentistry, have rarely been called upon to record the death of one whose loss will be more deeply lamented or more seriously felt.

*Resolved*, That in Dr. Austen's long connection with the Baltimore College of Dental Surgery, as one of its leading professors, he ably and successfully filled a high place of activity and usefulness, and discharged the duties entrusted to him with high honor and rare ability; and all the graduates of the Baltimore College, who sat under his instructions, and the dental profession everywhere, will ever remember with gratitude the labors of Prof. Austen, his numerous and valuable contributions to the science and literature of his profession, his personal inculcations as a teacher, and the deep interest he always manifested in his work.

*Resolved*, That as a Christian gentleman of learning and culture, he has left the testimony of a name and character that will endure so long as eminent services and faithfulness to duty shall be held as worthy of commemoration.

*Resolved*, That we tender our sincere and heartfelt sympathy to the family of the deceased in their heavy bereavement, and that a copy of these resolutions be presented to them, and a copy be entered upon the records of the Academy, and published in Dental Journals.

ELISHA G. TUCKER, *President*,
JACOB L. WILLIAMS, *Vice President*.
EDWARD N. HARRIS, *Cor. Secretary*.

